# Community Collectivism: A social dynamic approach to conceptualizing culture

**DOI:** 10.1371/journal.pone.0185725

**Published:** 2017-09-28

**Authors:** Birol Akkuş, Tom Postmes, Katherine Stroebe

**Affiliations:** 1 Faculty of Behavioural and Social Sciences, University of Groningen, Groningen, Netherlands; 2 Academy for Social Work, Saxion University of Applied Sciences, Enschede, Netherlands; Mälardalen University, SWEDEN

## Abstract

Culture shapes individuals, but the measurement of cultural differences has proven a challenge. Traditional measures of cultural values focus on individual perceptions. We suggest that values are established and maintained within social communities of proximate others, such as the family and its social environment. Within such communities, values serve to maintain collective harmony whilst preserving individual agency. From a social-dynamic analysis of communities, we infer that community values of loyalty regulate individual commitment, values of honor regulate norm compliance, and values of group hierarchy maintain a division of labor. In addition, communities may regulate the ways in which individuals have independent agency. A new scale to measure these values was validated in four studies (N = 398, 112, 465 and 111) among Dutch (religious and non-religious), Turkish-Dutch, Surinamese and Turkish groups. Values and practices were measured at the level of the individual (‘What do *you* value?’) and at the level of the perceived community (‘What does your *community* value?’). Results show that, unlike individual-level measures of individualism/collectivism, this scale has excellent reliability, differentiates between cultural groups, and has predictive validity for future (voting) behavior. This approach provides a new way of conceptualizing culture, a new measure of collectivism and new insights into the role of proximate others in shaping culture.

## Introduction

Cultural differences are undeniable: people from different cultural backgrounds act differently in a wide range of situations. But it is surprisingly difficult to pinpoint the source of these cultural differences and to measure their underlying causes in cognition and behavior. One reason why existing scales produce mixed results is that cultural difference is often assessed by assuming that “culture” at high level of aggregation (e.g., nations or “east vs. west”), shapes values of individuals. In this paper, we argue that measures of cultural values can be improved by taking into account the more proximate social environment in which people “do” culture. This social environment is often changeable and fluid, but the social relationships within it are relatively stable. This means that within-community cultural behavior is constrained not just by individual beliefs, but also by the (perceived) beliefs and actions of others in one’s cultural group [1], and in particular by those with whom one interacts frequently [2]. This also means that the social dynamics within communities shape the cultural behavior within it, and thus inform the content of the values that regulate within-community behavior.

Accordingly, this paper develops a social dynamic analysis of how values are established and maintained within communities, which can inform *how* to measure cultural values. This paper will argue that community collectivism is shaped by prevalent values and practices within the community of proximate others. This community environment (rather than abstract social categories such as nationality) should be the most proximate and primary source of cultural differences. This social dynamic analysis also helps to identify the content of the values most likely to be shaped within the community: the focus should be on values essential for regulating members’ community behavior (cf. [3]). Abiding by these values would be a prerequisite not just for the community to live harmoniously, but also for the individual to fit in and be accepted.

**Fig 1:**
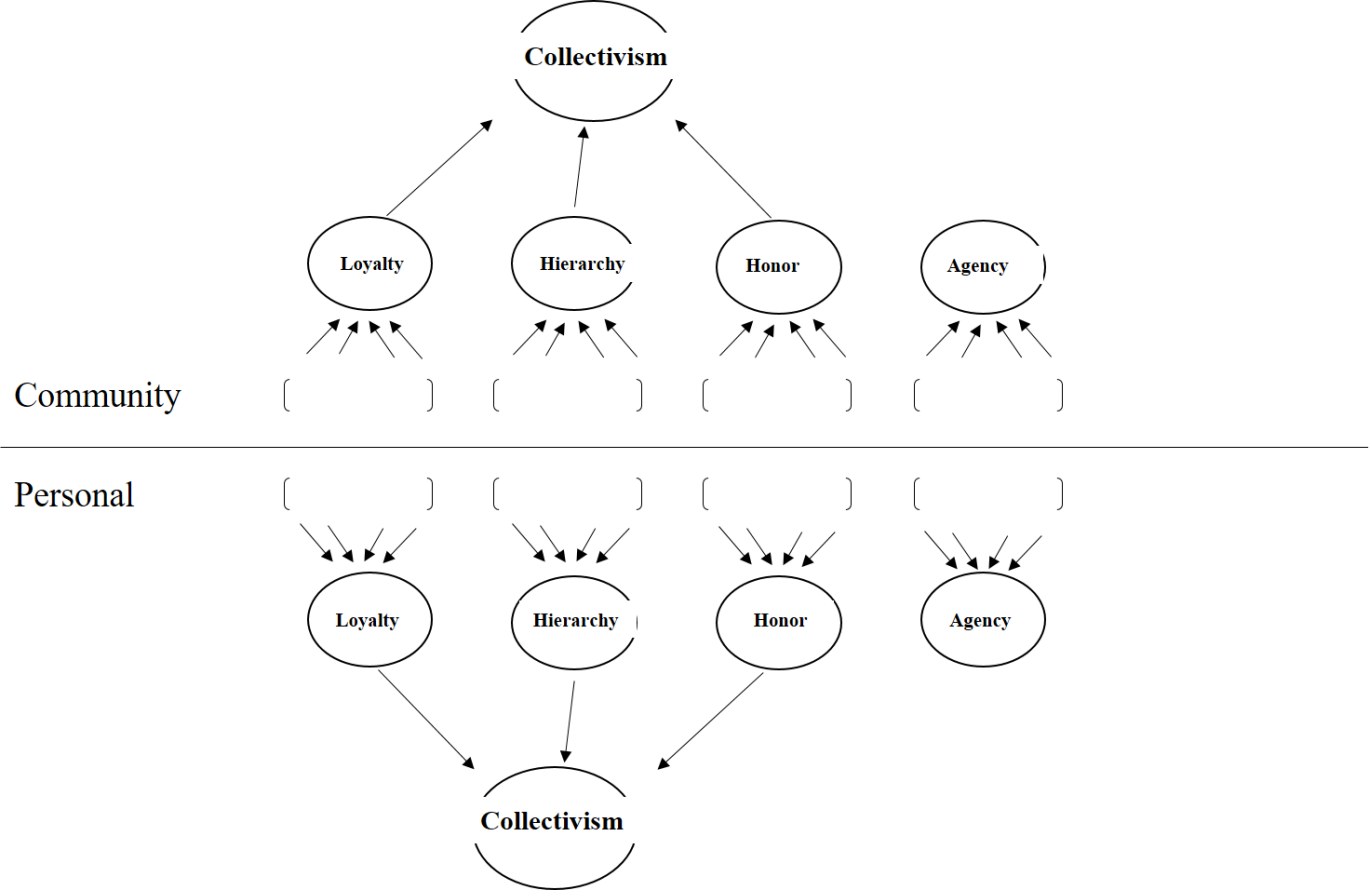
Structure of Community Collectivism.

The current paper contains an outline of this conceptual analysis, and we develop a measurement instrument from it. This is a measure of Community Collectivism that differs from existing measures in the form of measurement: we measure personal values and practices (personal collectivism or PerCol for short) as well as perceived values and practices within the community of proximate others (community collectivism, ComCol). This scale also differs in its content by focusing on values and practices that are essential to regulating the social behavior within the communities examined: issues of loyalty, honor, hierarchy and agency (Fig 1). Four studies are presented that put PerCol and ComCol to the test, and suggest that ComCol is distinctively effective.

### Culture: What is it and what does it do?

Although it has proven virtually impossible to define culture [4], one dominant definition is that cultures are ‘commonly shared meaning systems’ [5]. These meaning systems are typically translated to values [6, 7], such as those captured by the World Values Survey [8]. Together with the Individualism-Collectivism (Ind/Col)-heuristic (e.g., [9]), such values are among the most commonly used operationalizations of cultural differences. Although they are widely used, existing measures of such values have also been criticized as ill defined (e.g., [10]) and as being divorced from the social contexts within which individual thinking normally occurs (e.g., [11, 12]).

One of the debates regarding the conceptualization and operationalization of culture hinges on the question at what level culture and the values it is comprised of are supposed to be shared: at a national level, or rather the individual and situational level, or something in between? In defense of a national level approach, Hofstede & Bond [13] originally argued that distinctions between cultures would only be useful when comparing nations (see also [14]). Intuitively, the notion of national cultures rings true: in popular perception ‘national characters’ seem to exist. But empirically, the evidence is not so clear-cut. Terracciano and colleagues [15] compared in-group perceptions of national character with aggregated personality traits and concluded that perceptions of national character are “unfounded stereotypes” ([15], p.3). Similarly, for most ‘cultural’ values the within-country consensus is much lower than one would expect if values were indeed heavily influenced by culture *and* if cultures were a property of nations [10]. Fischer & Schwartz ([15], p.1137) conclude that “segments of the population emphasize different values because they have different experiences”: large within-country differences exist. Finally, it has been argued that cultural differences cannot be seen outside of the situational context they take place in: culture is primed or even determined by context (e.g., [16]).

One possible resolution to this issue is to make a distinction between personal and intersubjective values [1]. This suggests that people assess and perceive reality based on their sociocultural contexts: “rather than acting on their personal beliefs and values, people sometimes act on the beliefs and values they perceive to be widespread *in their culture*” ([1], p.482, emphasis added). These intersubjective perceptions of culture are likely to differentiate much better between cultural groups, and may indeed be consequential. The empirical work in this tradition tends to measure cultural values by reference to abstract social categories (e.g., Japanese, Poles, Americans) whose cognitive representations (like stereotypes) are likely to be intersubjective.

### Culture within communities

The National level may not be the only or best level at which intersubjective values should be measured. We argue that culture is likely to be most strongly and most consistently influenced by the community of concrete others with whom a person interacts on a day to day basis, such as one’s (extended) family. The national group (e.g., Americans) is a *depopulated* cognitive construct. Several studies have reported a ‘dissociation’ between personal and cultural values measured at this level (e.g., [1, 17–19]). The community one lives in is more concrete and known to the individual through personal experience. The community (e.g., family and extended family) is populated by others of flesh and blood. When acting within this setting or when expressing oneself, one has to take account of ongoing relationships and of their perceived values as inferred from, for example, past actions and manifested beliefs.

It is most likely within communities such as these that values are shaped, as they are requirements for coordinated social interaction, functioning and survival for *groups* [3,8]. Communities need particular mechanisms in order to regulate within-community behavior and to ensure the future preservation and integrity of the community. These mechanisms translate into values that are promoted within the community and that individual group members are supposed to live by. The same mechanism may operate within Nations or larger collectives, but their influence is likely to be much less direct.

If we want to examine what these values are, exactly, and how they are promulgated within the community, one could take different approaches. Like Schwartz’ Theory of Basic Individual Values, one could build a taxonomy of universal, personal values, some of which meet community requirements, while others meet individual or biological needs. But one could also take the community and the social dynamics within it as the starting point of such an analysis, because groups also have certain universal features. This is the approach we adopt here.

### Cultural values and their social dynamic origin

If we look for the fundamental features of groups that play a role in their formation and continuity, we may begin by distinguishing certain features of groups that appear to be universal in the sense that they can be found in all groups (and indeed across many species of social animal). All groups have a system of *care*, *roles*, and *rules*. By examining these systems and the social dynamics that sustain them, we can begin to identify the content of the shared cultural values that group members hold in common and that enable these systems to exist and function.

#### Care and communal cooperation: Community values of loyalty

Groups, especially those that are defined by close bonds, display a sense of unity (also referred to as entitativity, see [20]). From an evolutionary perspective, the propensity to form cooperative groups is seen as crucial in the survival and thriving of early hominids [21], and the desire to function in groups in order to be loved, valued and respected is considered an intrinsic property of humans (e.g., [22–24]). It is therefore not surprising that humans form groups that serve more than just instrumental goals: groups are the prime environment within which belonging and value is established, within which support and love is exchanged, in which cooperation can be expected. To enable this, groups should possess community values that encourage a certain amount of loyalty towards the group from all its members.

At the same time, there are substantial cultural differences in the degree of (un)conditionality and reciprocity of the loyalty that is expected within certain key groups such as the family (e.g., [25–27]). In some cultures, groups such as the family provide limited care, love and support (for example because care is institutionalized, because love is provided in friendship networks or because support is provided by the welfare state). Also, there are (most likely) substantial cross-cultural differences in the dimensions along which loyalty within particular groups is expected and disloyalty tolerated [28]. Overall, one would expect more interdependent or collectivistic cultural settings to be characterized by stronger awareness of community values that demand high levels of in-group loyalty. In other words, community values of loyalty should show distinct inter-cultural differences.

#### Roles and position: Community values of hierarchy

Another universal feature of human groups (as well as most animal groups) is that they have a degree of hierarchy and a division of roles. In strategic family therapy, for instance, a balanced and natural hierarchy between parents and children is emphasized as a necessity [29]. Within small groups, members enjoy having a clear division of roles which provide members with a sense of place and individual distinctiveness [30–32]. A 30-nation comparison of families by Georgas et al. [26] shows that some form of structure is a universal characteristic and, crucially, that these hierarchical structures are universally translated into (family) values. In sum, hierarchical structures appear to enable the functioning of *any* group. Consequently, all groups have a distinct set of community values pertaining to the hierarchical structure within the group that specify the responsibilities and privileges of position, that regulate interactions between positions and the rules for making the transition from one to the other.

Although the existence of hierarchies is ubiquitous, the way in which hierarchies are displayed and maintained appears to be quite variable between and within cultures. Some cultures have a distinctly “loose” approach to maintaining hierarchies in public settings, but in ‘tight’ countries (i.e. countries where norms are more strictly defined and there is little tolerance for norm violation) it is more likely that one finds autocratic forms of rule and a generic expectation that seniority, a form of hierarchy, is respected [33] (see also [13,34]). In sum, whilst all groups are characterized by some degree of internal organization or hierarchy, community values of hierarchy should show considerable inter-cultural differences.

#### Respect for rules and regulations: Community values of honor

Care and Roles can be seen as rules for how to behave. But these do not exert influence without a system that ensures rules are followed. It is often assumed that reward and punishment play a central role in this, but in practice group members only resort to these in extreme cases [35]. We propose that most of the time, the everyday behavior in communities is regulated by honor. All groups develop a communal understanding of what is ‘honorable’ and what is not. Similarly, in all groups, norm violation is shameful (even if it is not sanctioned, cf. [36]).

But even though norm violations are punishable in all groups and cultures, there is considerable variation in the degree of tolerance and the severity of the punishment [37]. There are also strong cultural differences in what counts as honorable and not. For example, Leung & Cohen [11] distinguish cultures of dignity, of honor, and of face. IJzerman & Cohen [38] even divide cultures into ‘honor cultures’ versus ‘nonhonor cultures’. Such cultural differences can appear extreme when comparing the cultural values that underpin so-called ‘honor killings’ (e.g., [39]) with the sexual libertarian values of some modern Western subcultures. But even at the so-called ‘nonhonor’ extreme, we find communities that promote the idea that it is virtuous to be industrious and hardworking (e.g., [40]), and shameful to be idle and unemployed (e.g., [41]). Thus, although the norms and sanctions may differ widely, we also see the same mechanism at work: the group prescribes what is virtuous and what not.

In sum, we regard honor as a means of enforcement against norm violation, and we want to disentangle this from the content of the virtues involved. Consequently, we distinguish cultures by the degree to which norm violations are monitored, and the possible punishment would threaten the status of individuals and/or the groups the norm violator belongs to.

#### Individuality: Having personal agency in a community

Values of Hierarchy, Honor and Loyalty are the backbones of a community’s system of roles, rules and care. But a communities’ members are also unique individuals with agency and a need for personal distinctiveness. The agency of individuals is traditionally considered a key difference between ‘Eastern’ and ‘Western’ cultures (e.g., [42, 43]) and as such should provide another means of discriminating between cultural groups. But there are good reasons why agency is qualitatively different from community-based regulatory values. Hierarchy, Honor and Loyalty are mechanisms that preserve *relatedness* [44–46]. By contrast agency preserves autonomy, a basic and universal need of individuals (see also: [47]). Agency may also be one way to achieve *distinctiveness*, which can be found as a motive to establishing identity in all cultures, albeit in very different ways across individualistic and collectivistic ones [48, 49].

In sum, personal agency may be part of the values of a group or community and can be considered another means to differentiate between cultures. But there are also suggestions that personal agency is an individual difference variable that is distinct from community-based values.

### Putting things together: Outlining a Community Collectivism Scale (CCS)

So far, we have identified four dimensions that are central to the social life of communities: Loyalty, Honor, Hierarchy, and Agency are all prerequisites for the community to live harmoniously, for individuals to fit in and be accepted. In line with the intersubjective approach, these four should not just be assessed by the individual’s personal beliefs about them. It is also important to know how community members perceive the beliefs and practices of *others* within their community. It is especially these perceived communal values that should predict culturally distinctive social behavior.

In constructing the scale, we had to ask ourselves what constitutes a ‘community’? There are many different ways of interpreting this term (e.g., one’s neighborhood or village, one’s peer group, etc.). Our conceptual approach looks at the influence of long-term relations on individual values and actions. It is widely accepted that for most people the central hub of socialization and the core relationships within which they anchor their values is the family (e.g., [44, 50, 51]). Indeed, a recent study confirmed that across many cultures, including supposedly individualistic ones, the most important group in people’s lives were nuclear and extended family [52]. The family, in turn, is embedded in a network of families (e.g., at schools or in neighborhoods) who reference each other (e.g., [46, 53]). Therefore, within the CCS, we operationalized community as the core and extended family, within a wider network of families seen as in-group.

It is important to be clear about how this approach departs from other methods of operationalizing cultural difference. CCS differs from the most often used measures of Individualism/Collectivism such as INDCOL95 [9] and from the intersubjective approach in several ways. First, CCS uniquely distinguishes social dynamics of loyalty, hierarchy, honor and agency. Compared with INDCOL95, CCS distinguishes community and personal values. Compared with the intersubjective approach, CCS is unique in its focus on the community rather than higher-order groups such as the Nation.

It is also important to highlight differences with Schwartz’ values scale. Schwartz distinguishes *motivational* values that individual group members are supposed to live by. Accordingly, the Schwartz Values Survey (SVS) asks respondents to rate values (e.g., honoring of parents and elders; showing respect) for their importance “as a guiding principle in my life”. Schwartz’ scale is thus, ultimately, a scale that minutely dissects all the values that individuals can possibly possess. By comparison, the CCS is much more situated and focused on a specific set of systemic characteristics of *groups* (hierarchy, norms, loyalty, agency). Although inevitably there is some overlap in content between these four and Schwartz’s system of values (which after all purports to catalogue all of them) there are marked and striking differences in approach. CCS does not just aim to measure personal values: Community values are promulgated through practices and beliefs as well: what matters is how culture is *done* within the *community*. By contrast, Schwartz’ approach is most interested in how individuals see *themselves*.

In sum, we developed a 4-dimensional Community Collectivism Scale (CCS), assessing values of Loyalty, Hierarchy, Honor and Agency. Values were measured at the personal level (the individual’s own views and practices) and the collective/communal level (the perceived views and practices of others). We conducted four studies in order to: i) assess the scale’s methodological characteristics and validity, and determine the definitive version, ii) assess and confirm the ability to discriminate between cultural groups, iii) assess the types of behaviors and attitudes the scale can predict, and iv) assess how CCS related to existing measures. The results are discussed in the following sections.

## Method and results

### Samples

We tested the CCS in four samples: Sample 1 consisted of 398 participants, of which were 183 of Turkish descent (44% women, M_age_ = 31), and 215 indigenous Dutch (46% women, M_age_ = 46), all inhabitants of the Netherlands and members of an online panel (PanelClix). We compared the cultural groups on demographical variables (age, sex, occupation and education). Of the Turkish sample 25,7% were students, whereas only 3,7% of the indigenous Dutch sample were. With the exception of age there were no further significant differences (M_indigenous Dutch_ = 46); M_Turkish_ = 31). Fourteen cases were considered multivariate outliers (Mahalanobis distance, p < .001), and removed. Sample 2 were 112 PanelClix-members who also participated in the first wave of data collection, of which 55 of Turkish descent (49% women, M_age_ = 34), and 57 indigenous Dutch (49% women, M_age_ = 49).

Sample 3 were 465 Panelclix-members from four cultural groups (all inhabitants of the Netherlands): (a) non-religious indigenous Dutch (n = 127, 47% women, M_age_ = 39), (b) orthodox protestant indigenous Dutch (n = 124, 65% women, M_age_ = 39), (c) of Turkish descent (n = 94, 53% women, M_age_ = 31) and (d) of Hindustani Surinamese descent (n = 120, 67% women, M_age_ = 37.5). We compared the cultural groups on demographical variables (age, sex, occupation and education). Gender was not evenly distributed with the orthodox-protestant Dutch (65% women) and Hindustani Surinamese (67% women) sub-samples. The Turkish Dutch sample was less likely to have higher education than the other groups and had a significantly lower mean age (M_non-relig.Dutch_ = 39, M_orth-prot.Dutch_ = 39, M_Hind.Sur._ = 38, M_Turkish_ = 31). Eight cases were identified as multivariate outliers (Mahalanobis distance, p < .001), and removed.

Finally, Sample 4 were inhabitants of Turkey who were part of a Qualtrics panel. Sample 4 (N = 111, 44% women, M_age_ = 39) participated in a two-wave study (first measurement March 2014, second October 2015).

#### Outline of the studies

Our goal was the construction and validation of a scale that gauges cultural differences, and predicts differences in attitudes and behaviors. Construction and validation of CCS was conducted in the following stages: The first stage consisted of item generation based on our proposed structure (four dimensions at the community and personal level). We then reduced the number of items for each hypothesized dimension, retaining the items most suitable for further analysis (Sample 1). Subsequently, factor analysis across hypothesized dimensions was conducted to ascertain factor structure at the personal and community level (Samples 1 and 3), followed by bifactor analyses in order to explore whether our dimensions are better explained by one underlying (cultural) factor (Sample 1). We then established whether the definitive scale meets the basic criteria for measurement invariance. Also, we determined validity by comparing CCS with existing scales (Samples 1 and 3), looking at whether it differentiates between cultural groups (Samples 1, 2 and 3), whether it predicts attitudes (toward family, kin and work, Sample 1) and behavioral orientations (measured via behavioral scenarios across the different dimensions, Samples 1,2 and 3). Finally, to study the predictive behavior of the scale for behavioral outcomes, we report results of a longitudinal study in which participants complete the CCS at time 1, and report on recent behavior (voting, social conflicts) at time 2, one and a half years later (Sample 4).

#### Choice of samples

The choice of sample was guided by the need to (a) differentiate between cultural or subcultural groups that differ in Individualism-Collectivism whilst (b) keeping the language within which the test was administered constant. Consistent with our group dynamic approach, the idea that comunities shape values implies that culture and cultural values can be distinguished within and not just between nations. We thus searched for groups that all use the same language but have different community structures for cultural or subcultural reasons.

In the first three studies, we compared different subcultural groups in the Netherlands which meet these criteria. Indigenous Dutch culture is traditionally considered to be individualistic (e.g., [54] Van Oudenhoven, 2001), and could therefore be contrasted with the culture of a collectivist immigrant group in The Netherlands, the Turks (e.g., [55, 56]). Samples 1 and 2 therefore compared these groups. Sample 3 was approached for a study that aimed to further refine our scale, as well as establish whether it can also differentiate between (sub)cultural groups. Sample 3 therefore also compared religious subcultures: secular Dutch versus strict-protestant Dutch. This is important to establish that between-community differences need not be ethnic in origin. Compared with secular Dutch, strict-protestants display a distinctly different community structure (e.g., as evidenced by a birth ratio that is almost double the national average, a tendency to live in close proximity to same-faith others in a specific “bible belt” region of the country, strong community ties, etc.). Sample 3 also sought to generalize findings from Turkish Dutch to Hindustani Surinamese Dutch. The Hindustani Surinamese are migrants (and their descendants) from the former Dutch colonies of Surinam who originally come from India; they are considered to be culturally collectivistic (e.g., [57]). A further advantage of using Turkish and Hindustani (Surinamese) cultures as collectivist references, is the fact that these cultures are often overlooked in the Ind/Col-research, where generally East-Asian and Northern American cultures are contrasted. Sample 4, finally, consisted of inhabitants of Turkey, all indigenous Turkish. Inhabitants of this country are interesting because of the stark differences between modern secular Turkish communities and more traditionalist ones; this allows us to test CCS’ ability to differentiate between communities within a culture.

#### Ethics statement

Participants were members of an online panel and were recruited by the panel provider (Panelclix). All participants were of the age of 18 or older. This was checked by both the panel provider and ourselves (via a question in the questionnaire). All participants were asked their explicit consent to participate in filling out a questionnaire about social relations before they started our (online) questionnaire. It was also explicitly pointed out that they could stop and cancel their participation at any time, and that their responses would be treated confidentially. They could give or deny consent by either clicking the button that explicitly stated they gave their consent, or the button that explicitly stated that they did not wish to continue. In the latter case, the questionnaire would be aborted immediately and the participant would be thanked for their input. If the participant did consent, the questionnaire would commence, and they were given disclosure about the intent of our research when finishing the questionnaire. This whole procedure was submitted to and approved by the Ethical Committee (Psychology) of the University of Groningen.

## Results: Scale construction

### How were items generated?

For each of the four hypothesized dimensions (Honor, Agency, Loyalty, Social Hierarchy), approximately twelve items were construed that could be administered at the Community and Personal level. Some items were adapted from existing scales such as the INDCOL95 [9] and the World Values Survey [58]. We aimed to capture a broad spectrum of the facets of each dimension. This procedure resulted in a total of 94 items (47 items mirrored on Community and Personal levels) that were assessed on scales ranging from 1- (strongly disagree) to 5 - (strongly agree). The resulting scale consisted of two separate blocks of Personal and Community items. Community items were preceded by an explanation of the concept of community saying: “Some of the statements that will be presented to you refer to your community. With your community, we mean your core family, your extended family and other families (in your environment) that matter to you”. The reference to “other families that matter to you” was included so that participants would focus on the ingroup context of their families, and be less likely to make contrastive social comparisons to families of the “outgroup”. We included a check for participants to confirm that they had read and understood this.

### Which items were selected?

Item selection (Sample 1) was based on statistical considerations as well as item content, in conjunction with theoretical considerations. We inspected inter-item and item-total correlations as well as conducting exploratory factor analyses for each dimension separately [59, 60]. In order to maximize validity, we balanced the need for internal consistency with adequate heterogeneity of item content. Redundant items (*r* > .8), as well as items whose item-total correlation were too small (*r* < .4), were filtered out. Where the characteristics of items conflicted between the levels, priority was given to the Community subscales (i.e., an item loading high on the personal but not community level would be eliminated). We conducted exploratory factor analyses for each dimension separately with Sample 1 [59, 60] and items with loadings lower than .4 were eliminated. As a last criterion, items with communalities < .3 were removed. With this procedure, we retained three to four items per subscale. We aimed to retain negatively as well as positively worded items, in order to check for and prevent acquiescence effects. However, none of the negatively worded items passed the criteria. As a result, we retained 15 items mirrored at the Community and the Personal level (30 items in total) that formed reliable subscales (Table 1).

**Table 1:** Community Collectivism Scale–selected items with factor loadings and scale reliability.

Level	Dimension	Item	Subscale α	Factors			
Community				1	2	3	4
	Honor		.80				
		HC1: In my community it is considered a disgrace if there is gossip about you.		**.54**			
		HC2: In my community, honor is the most important thing for people.		**.80**			
		HC3: Our community monitors if people observe the unwritten rules.		**.60**			
		HC4: In my community, members of the family feel responsible for preserving and protecting another family member’s honor.		**.84**			
	Agency		.67				
		AC1: In my community, you are responsible for the important choices in your life					**.76**
		AC2: In my community, everyone is responsible for their own life.					**.60**
		AC3: In my community striving for autonomy is considered good.					**.60**
	Loyalty		.74				
		LC1: In my community people experience the problems of their family members as if they were their own problems.				**.45**	
		LC2: In my community family ties are very strong.				**.65**	
		LC3: People are expected to support their family members, even if they do not want to.				**.53**	
		LC4: In my community you are expected to do what you can when a family member needs you.				**.74**	
	Hierarchy		.82				
		SHC1: In my community, it is generally believed that men have a more important voice than women.			**.61**	-.26	
		SHC2: In my community, your elders’ opinions are more important than your own opinions are.			**.95**		
		SHC3: In my community people believe that older people have a higher status than the young.			**.81**		
		SHC4: In my community you are expected to accept that some people in your family have more to say, and others less.			**.44**		
**Personal**							
	Honor		.77				
		HP1 I would consider it a disgrace if there would be gossip about me.		**.66**			
		HP2: Honor is the most important thing for me.		**.73**			
		HP3: I monitor if people (from my community) observe the unwritten rules.		**.62**			
		HP4: I feel responsible for preserving and protecting my family member’s honor		**.71**			
	Agency		.72				
		AP1: I am responsible for the important choices in my life.					**.74**
		AP2: I am responsible for my own life.					**.85**
		AP3: I consider striving for autonomy as good.					**.54**
	Loyalty		.73				
		LP1: I experience the problems of family members as if they were my own problems.			**.47**		
		LP2: My ties with my family are very strong.			**.77**		
		LP3: I would support my family members, even if I wouldn’t want to.			**.63**		
		LP4: I will do what I can when a family member needs me.			**.60**		
	Hierarchy		.74				
		SHP1: I believe men should have a more important voice than women.			-.28	.38	
		SHP2: My elders’ opinions are more important to me than my own opinions are.				**.79**	
		SHP3: I think that older people have a higher status than the young.				**.86**	
		SHP4: I accept that certain people in my family have more to say than others				**.46**	

*Note*: N = 393; Principal Axis Factoring with Promax-rotation: pattern matrix; Only factor loadings > .25 are shown, factor loadings > .40 are in boldface.

### What is the factor structure?

We aimed to ascertain the hypothesized four-dimensional structure, first with Sample 1, subsequently with Samples 2 and 3. Because we assume Community Collectivism to be a broad construct we expected high factor loadings to be rare. Furthermore, because at the collective level in particular the 4 dimensions would be interconnected we also expected some cross-loadings. In other words, given the theoretically derived item generation and item selection strategy it would be *problematic* to find a completely orthogonal factor structure. We performed exploratory factor analysis via Principal Axis Factoring (with Promax rotation) for the community versus personal level separately. Note that this approach (subscales first, then superordinate structure) is conservative with respect to our hypotheses. With regard to the community level we determined the number of factors as four via a parallel analysis on the scree-plot [60] (Russel, 2002). These four factors corresponded well with the four hypothesized dimensions Hierarchy, Loyalty, Honor and Agency (Table 1). We found only one substantial cross loading (i.e. >.25) between dimensions, with SHC1 (Social Hierarchy Community level, item 1) loading -.26 on the Loyalty dimension. Furthermore, the loading of item SHC2 (Social Hierarchy Community level, item 2) was relatively high (.95).

At the personal level, we also extracted 4 factors. Again, the factor structure was as expected. The order in which factors were extracted differed slightly from the Community level: Honor was extracted first at this level as well, but Loyalty and Hierarchy changed places, while Agency again was extracted last (see Table 1). The only substantial cross-loading was SHP1 (Social Hierarchy Personal level, item 1), which cross-loaded (-.28) on the Loyalty dimension. In order to confirm the factor structure, we also performed the factor analyses on Sample 3. The factor structure that emerged for Sample 1, re-emerged on both levels.

Of the four dimensions, the Agency dimension was the weakest at both levels across both samples. Thus, the concept of agency appears to be rather poorly defined as a distinct factor. Moreover, the factors Hierarchy, Loyalty and Honor are more strongly mutually correlated, than they are with Agency.

To further clarify the factor structure, we conducted a confirmatory factor analysis (CFA) with Satorra-Bentler correction, using the Lavaan-package (0.5–15) for R [61] (Rosseel, 2012). CFA of a four-factor model for the Community level indicated a good fit (corrected for 4 cross loadings): χ^2^ (80) = 120.14, *p* = .002, CFI = .975, RMSEA = .036, and SRMR = .039. Fit of the four-factor model at the Personal level was acceptable (corrected for 6 cross loadings): χ ^2^ (80) = 161.50, *p* < .001, CFI = .946, RMSEA = .052, and SRMR = 0.047.

Similar results were found in a confirmatory factor analysis for Sample 3: CFA of a four-factor model for the Community level indicated a good fit (corrected for 4 cross loadings): χ^2^(80) = 126.15, *p* = .001, CFI = .971, RMSEA = .038 and SRMR = .038. Fit for the four factor model at the Personal level was again acceptable (corrected for 4 cross loadings): χ^2^ (80) = 152.90, *p* < .001, CFI = .950, RMSEA = .047 and SRMR = .047.

In sum, the findings are consistent with our hypothesized factor structure. Moreover, solutions at the Community and Personal levels were somewhat different. This supports the distinction between cultural norms at the Community versus Personal level, underscoring the necessity to measure these levels separately, especially in the case of the Honor, Loyalty and Hierarchy dimensions, as the order of extraction may well reflect differences in the centrality of these dimensions.

### Bifactor analysis

We then assessed whether at the Community level, the variance in the sample is explained by a single underlying (cultural) dimension, a Community factor that encompasses the dimensions of loyalty, hierarchy and honor. In order to do so we conducted bifactor analysis [62, 63] on Sample 1 using the Lavaan-package for R. We specified two models, a model with all four factors and a model with only the three specific Community factors (Honor, Loyalty and Hierarchy). Both models also specified a general factor predicted by *all* items. We applied the Schmid-Leiman orthogonalization and thus assumed all factors to be uncorrelated [63]. Due to the ‘beta’ nature of the Lavaan R-package (05–15), the forced orthogonalization of the factors did produce some computational errors, which we managed to circumvent by fixing item HC2 on 0.3. This being the most conservative solution, as it maximizes the amount of variance explained by the Honor factor.

The (confirmatory) bifactor analysis with Satorra-Bentler correction for the four-factor model at the Community-level reveals a workable fit (specifying four cross-loadings): χ^2^(72) = 144.29, *p* < .001, CF = .954, RMSEA = .051 and SRMR = .053. However, a closer examination of the factor loadings on the general factor (*g*) reveals that in contrast to the other three dimensions there is little relation between *g* and the three Agency items (loadings varying between -.14 and -.10). A three-factor model excluding Agency considerably improved the fit of the model (corrected for three cross loadings): χ^2^(40) = 80.3, *p* < .001, CFI = .972, RMSEA = .051 and SRMR = .039. Importantly, this bifactor analysis allows us to assess the strength of the underlying dimension (the general factor) relative to each individual component. The results of this analysis are provided in Table 2. As can be seen, the general factor explains 56% of all variance (for the three-factor model). Each component explains some additional variance, ranging from 18% to 9%. This implies that there is merit in differentiating between Community-level Hierarchy, Loyalty, and Honor, but that a general ‘Community’ level factor also explains substantial variance across all three.

**Table 2:** Explained variance for factor structure alternatives.

Factor structure	Factors	Community	Personal
**Bifactor: 4 factors**			
	Honor	8%	14%
	Loyalty	15%	13%
	Hierarchy	13%	16%
	Agency	15%	15%
	g	49%	43%
**Bifactor: 3 factors**			
	Honor	9%	11%
	Loyalty	18%	21%
	Hierarchy	17%	16%
	g	56%	52%

*Note*: N: 393

On the basis of these findings, we took the means of the scores of the Honor, Loyalty and Hierarchy subscales for each level as general indexes of ComCol (Community Collectivism score) and PerCol (Personal Collectivism score). Agency is most likely to be seen as a separate factor, and is included in the analyses as such. The correlation matrix in Table 3 confirms this assertion.

**Table 3:** Correlations of CCS-dimensions and indexes.

		Community					Personal				
		Honor	Loyalty	Hierarchy	Agency	Collectivism	Honor	Loyalty	Hierarchy	Agency	Collectivism
**Community**	Honor	1	.531**	.568**	.045	.871**	.689**	.369**	.453**	.088	.663**
	Loyalty	-	1	.374**	.284**	.747**	.432**	.693**	.216**	.324**	.562**
	Hierarchy	-	-	1	-.142**	.817**	.427**	.225**	.692**	-.089	.592**
	Agency	-	-	-	1	.061	-.067	.268**	-.243**	.884**	-.037
	Collectivism	-	-	-	-	1	.637**	.507**	.575**	.117*	.746**
**Personal**	Honor	-	-	-	-	-	1	.430**	.533**	.003	.867**
	Loyalty	-	-	-	-	-	-	1	.192**	.320**	.668**
	Hierarchy	-	-	-	-	-	-	-	1	-.183**	.766**
	Agency	-	-	-	-	-	-	-	-	1	.043
	Collectivism	-	-	-	-	-	-	-	-	-	1

*Note*: n: 398 (Sample 1)

*: p < .05

**: p < .01.

### Measurement invariance

A basic premise for using a scale to compare groups, is the ability of the scale to measure the construct regardless of group characteristics that are not relevant within that context [64–67]. We analyzed our models (the four factor models separately for both levels) for the two groups in Sample 1 (indigenous Dutch and Dutch of Turkish descent), to examine configural, metric and scalar invariance. We did not consider full scalar invariance (also constraining means) as the purpose of the scale is to measure differences between groups. Specifically, we tested the Goodness of Fit indexes (CFI, χ^2^/df-ratio and RMSEA) when constraining the factor loadings (metric invariance) and intercepts (scalar invariance) for all groups. We performed (I) a Multi Group Confirmative Factor Analysis (MGCFA) to establish configural invariance, (II) then constrained factor loadings to analyze metric invariance, and finally (III) also constrained intercepts to analyze scalar invariance, correcting for cross-loadings and with Satorra-Bentler adjustment, and using R with the Lavaan and semTools packages.

We analyzed the results following the recommendations by Chen [64], and applying the proposed criteria, i.e. a decline of CFI of less than .10 and an increase of RMSEA of less than .015 when constraining the factor loadings would indicate non-invariance at this level. Subsequently, the Goodness of Fit should not decline more than ΔCFI≤ -.01 and ΔRMSEA≥ .015 when also constraining the intercepts.

The MGCFA of both models confirmed acceptable fit, with CFI = .952 and RMSEA = .045 (Collective model) and CFI = .942 and RMSEA = .053 (Personal model). When constraining factor loadings, the decline in Goodness of Fit indices met Chen’s criteria for both models: ΔCFI was .007 for the C-model and .001 for the P-model, ΔRMSEA was .001 (C-model) and .004 (P-model), while the χ^2^/df-ratio remained below 3 for both models. Further constraining the intercepts resulted in mixed results. Criteria were met for the C-model, with ΔCFI = -.009 (total CFI = .938), ΔRMSEA = .001 (total RMSEA = .047). However, criteria were only partially met for the P-model. Criteria for CFI were not met: ΔCFI = -.038 (total CFI = .905). Criteria for RMSEA were met: ΔRMSEA = .013, total RMSEA = .062. Again, the χ^2^/df-ratio remained below 3 for both models.

In sum, configural and metric invariance were fully confirmed, while scalar invariance was partly. Only the decline in CFI for the P-model for scalar invariance did not meet criteria, all other parameters suggested invariance criteria were met. Mindful of Chen’s [64] final remarks, that these criteria should be used with caution, and the fact that CCS consists of the combination of both Collective and Personal levels, we conclude that the criteria for invariance are largely met, and therefore the comparisons between groups based on CCS-scores reported below, are very likely meaningful and valid.

## Results: Validation

### How does CCS relate to existing measures?

In order to assess the extent to which CCS can be considered distinct from a traditional measure of individualism/collectivism we assessed the correlations between the (reduced) INDCOL95 [68] and CCS (see Table 4). Results confirm our expectations. Correlations between INDCOL95 and the CCS subscales range from .20 (Agency Community) to .45 (Honor Personal). The ComCol index (the average of Honor, Loyalty and Hierarchy at the Community level) and PerCol (the average of Honor, Loyalty and Hierarchy at the Personal level) correlate respectively .48 and .51 with INDCOL’95. Therefore, we can conclude that although CCS and INDCOL’95 converge to some extent, they also tap into distinct enough constructs, as is to be desired from a scale that supplements an existing construct.

**Table 4:** Correlations between INDCOL95 and Community Collectivism subscales.

Scale	CCS		Loyalty		Hierarchy		Honor		Agency	
	ComCol	Per Col	Com	Per	Com	Per	Com	Per	Com	Per
INDCOL95	.48**	.51**	.38**	.37**	.38**	.36**	.42**	.45**	.20**	.31**
Horizontal Collectivism	.33**	.39**	.32**	.36**	.18**	.19**	.33**	.35**	.21**	.30**
Vertical Collectivism	.49**	.59**	.32**	.35**	.42**	.49**	.43**	.51**	-.14**	-.08
Horizontal Individualism	.04	-.03	.08	.04	.02	-.07	-.01	-.04	.40**	.45**
Vertical Individualism	.38**	.35**	.26**	.20**	.34**	.29**	.33**	.31**	.13*	.24**

*Note*: n: 390 (Sample 1)

**: p < .01

*: p < .05. Com: Community-level, Per: Personal-level.

We also tested (in Samples 2 and 3) how CCS relates to Diener’s Subjective Life-Satisfaction Scale [69] (5-item), and the Rosenberg Self-Esteem Scale [70] (10-item). We expected that higher scores on Hierarchy (and possibly Honor) would negatively correlate with Life Satisfaction and Self Esteem, whereas higher scores on Loyalty and Agency were expected to positively correlate with both. We reasoned that higher degrees of Hierarchy and Honor would constitute a stricter structure and more control and as such might limit individuals. Conversely, a higher degree of Loyalty would imply that an individual feels more support from his community and is inclined to return support, whereas a higher degree of Agency would imply that the individual feels more in control of their life and fate.

The correlations between CCS subscales and these measures are presented in Table 5. What we find, is that Self Esteem correlates weakly to moderately across all dimensions, as well as the ComCol and PerCol indexes. The correlations with Agency Personal (*r* = .35, *p* < .001) and Hierarchy Personal (*r* = -.33, *p* < .001) stand out however. This corresponds with our expectations: people who feel they are more in control of their fate have higher self-esteem, whereas people who are more willing to accept a (strict) hierarchy rate themselves lower. Correlations with Diener’s Subjective Life Satisfaction Scale are low or non-significant, but do, as expected, point to a weak positive correlation with the Agency and Loyalty subscales. And again: higher agency would mean a higher belief in control over one’s life and more satisfaction as a consequence. A higher score on Loyalty in CCS would imply that a person is more willing to provide and receive support, thus feeling more satisfied with life. Overall the pattern of correlations between CCS and the measures of Self Esteem and Life Satisfaction was as expected.

**Table 5:** Correlations between Community Collectivism subscales and Self-Esteem, and Life-Satisfaction measures.

Scale	CCS		Loyalty		Hierarchy		Honor		Agency	
	Com Col	Per Col	Com	Per	Com	Per	Com	Per	Com	Per
Self Esteem	-.09	-.18**	.17**	.15**	.-.21**	-.33**	-.14**	-.16**	.17**	.35**
Life Satisfaction	.05	.04	.16**	.17**	-.03	-.05	.01	.05	.18**	.16**

*Note*: n: 414 (Sample 3)

**: p < .01. Com: Community-level, Per: Personal-level.

### Does CCS explain between-group differences?

In order to assess whether CCS distinguishes between cultural groups we compared the following groups: Sample 1 compared indigenous Dutch and Turkish Dutch, Sample 3 compared non-religious indigenous Dutch, orthodox-protestant indigenous Dutch, Turkish Dutch and Hindustani Surinamese Dutch.

Comparing the means for Sample 1 (Table 6) we see that means are significantly higher for the Turkish group than the indigenous Dutch group on three of the four subscales (Honor, Loyalty and Hierarchy), as well as on both ComCol and PerCol. The exception is the Agency scale, for which there is no significant difference either at individual or Community level. The effect sizes (Cohen’s *d*) of the differences between both groups show that group (cultural) differences between Dutch and Turkish are substantially larger at the Community level (see Table 5). At the Community level the effect sizes of the first three dimensions range from 1.00 (Hierarchy) to .52 (Loyalty), whereas at the Personal level they range from .66 (Hierarchy) to .32 (Loyalty). However, the Agency subscale again shows a different pattern: the effect size on the Community level is .11, but the direction of the effect is reversed: the Dutch mean is slightly higher. On the Personal-level there are virtually no cultural differences (*d* = .05). Clearly Agency does not discriminate between these two groups. Importantly, the effect sizes for CCS subscales, as well as for both indexes ComCol (1.03) and PerCol (.69) are considerably larger than those of the (reduced) INDCOL’95 (.41 for the scale). This shows that CCS does a much better job at discriminating cultural differences than the INDCOL’95.

**Table 6:** Means (SD) and effect sizes (Cohen’s d) of a priori dimensions and aggregate indexes of CCS and INDCOL’95 dimensions.

Scale	Factor	Level	NL	TR	Effect Size
**CCS**					
	Honor				
		Community	2.83 ^a^ (.66)	3.56^b^ (.71)	.94
		Personal	2.88 ^a^ (.71)	3.33^b^ (.73)	.60
	Loyalty				
		Community	3.33 ^a^ (.59)	3.66^b^ (.63)	.52
		Personal	3.54 ^a^ (.58)	3.74^b^ (.64)	.32
	Hierarchy				
		Community	2.44 ^a^ (.70)	3.23^b^ (.64)	1.00
		Personal	2.46 ^a^ (.66)	2.93^b^ (.70)	.66
	Agency				
		Community	3.99 ^a^ (.48)	3.93^a^ (.60)	-.11
		Personal	3.77 ^a^ (.47)	3.80^a^ (.56)	.05
	ComCol		2.87 ^a^ (.49)	3.48^b^ (.53)	1.03
	PerCol		2.96 ^a^ (.48)	3.34^b^ (.54)	.69
**INDCOL**					
	IndCol		3.21^a^ (.37)	3.37^b^ (.41)	.41
	Horizontal Collectivism		3.41^a^ (.53)	3.51^a^ (.51)	.19
	Vertical Collectivism		2.83^a^ (.56)	3.08^b^ (.64)	.41
	Horizontal Individualism		3.55^a^ (.61)	3.58^a^ (.67)	.05
	Vertical Individualism		3.10^a^ (.66)	3.36^b^ (.56)	.41

*Note*: NL: indigenous Dutch (n: 215), TR: of Turkish descent (n: 183); Negative effect sizes indicate that indigenous Dutch mean is higher than mean of participants of Turkish descent. Subscripts with different indices differ at (p < .05)

We also examined whether CCS distinguishes between groups in Sample 3 (i.e., orthodox-protestant indigenous Dutch, Turks and Hindustani-Surinamese compared to non-religious indigenous Dutch; Table 7). Again, we found evidence that the CCS distinguishes (sub)cultural groups well. For the Turkish and Hindustani-Surinamese the largest differences were, as in Sample 1, found for Hierarchy and Honor. Not unexpectedly, the pattern of results differed somewhat for the orthodox-protestants where greatest differences were found for Loyalty and the Community level of Agency.

**Table 7:** CCS means and effect sizes of non-religious Dutch vs other groups on Community Collectivism Scale.

Factor	Level	Non-religious Dutch	prot. Dutch		Turks		Hindustani	
		M (SD)	M (SD)	Effect Size	M (SD)	Effect Size	M (SD)	Effect Size
Honor								
	Community	2.82 (.74)	3.08** (.70)	.35	3.51** (.71)	.93	3.35** (.75)	.78
	Personal	2.86 (.75)	3.01 (.72)	.20	3.32** (.76)	.62	3.14** (.79)	.38
Loyalty								
	Community	3.33 (.54)	3.66** (.59)	.61	3.74** (.73)	.75	3.63** (.78)	.55
	Personal	3.43 (.66)	3.83** (.58)	.60	3.76** (.75)	.50	3.65* (.76)	.32
Hierarchy								
	Community	2.34 (.85)	2.41 (.78)	.09	3.22** (.80)	1.04	3.12** (.84)	.92
	Personal	2.26 (.86)	2.48* (.76)	.25	2.93** (.97)	.78	2.66** (.77)	.46
Agency								
	Community	3.83 (.63)	3.45** (.63)	-.59	3.53** (.72)	-.47	3.57** (.62)	-.40
	Personal	4.12 (.64)	3.90** (.51)	-.35	3.86** (.69)	-.41	4.15 (.72)	.04
ComCol		2.82 (.56)	3.04** (.49)	.39	3.48** (.60)	1.17	3.38** (.61)	1.00
PerCol		2.85 (.57)	3.11** (.48)	.45	3.32** (.60)	.83	3.16** (.58)	.55

*Note*: Mean difference significance is indicated by *: p < .05 and **: p < .01; Cohen’s D effect size with non-religious indigenous Dutch (n = 118) as reference group (mean and sd) versus orthodox-protestant indigenous Dutch (n = 116), of Turkish descent (n = 81) and of Hindustani Surinamese descent (n = 107); Negative effect sizes indicate that non-religious indigenous Dutch mean is higher than mean of participants of compared group.

We note that although Agency did not discriminate between indigenous Dutch versus Turkish Dutch in Sample 1, our findings for Sample 3 suggest that this dimension does uncover group-cultural differences in general. Apparently, the groups compared in Sample 1 (indigenous Dutch and Dutch of Turkish descent) happen to share similar values with regard to this dimension.

Overall, we can conclude that CCS discriminates very well between cultural groups (indigenous Dutch compared to Turkish and Hindustani-Surinamese), as well as between sub-cultural groups (non-religious indigenous Dutch compared to orthodox protestant indigenous Dutch), and across all dimensions.

### What behaviors does CCS predict?

We assessed whether CCS’s predicts attitudes and behaviors with 20 scenarios (two in Sample 1, 18 in Samples 2 and 3) in which cultural differences were expected to surface. The scenarios were devised following the expectation that higher scores on Loyalty, Hierarchy and Honor would predict behaviors and attitudes prioritizing group interests versus personal interests. We expected that Agency would predict the opposite. The rationale behind these expectations was that cultural orientations should show in such everyday events that touch on social relations. Within the context of CCS, the value domains of Loyalty, Hierarchy and Honor have the function to regulate the community. Scoring higher on these values, should therefore predict attitudes and behaviors that reflect prioritizing group interests above personal interests. While Agency is not the opposite of this, scoring higher on this dimension may predict attitudes reflecting greater margins for individual ‘deviation’. However, as Agency also includes an element of ‘greater personal responsibility’, we expected that the outcomes with regard to this value domain could turn out less straightforward than the outcomes with regard to the other three value domains.

We developed 18 short scenarios (all scenarios and analyses are available from the first author) in Samples 2 and 3 that were centered on the CCS-dimensions and on possible transgressions of the underlying cultural values, both in a neutral social context and in a context that involved medical or psychosocial healthcare. Furthermore, we were interested in exploring how participants thought their close relatives would act. Participants were presented with three possible responses that were formulated to be either increasingly prioritizing group interests, or increasingly prioritizing individual interests. An example of a scenario would be: ‘*Since your father in-law passed away*, *your mother in-law lives with you and your partner*. *This has given rise to some tensions*, *for which you have sought counseling*. *Your therapist advises you to ask your mother in-law to consider moving to an assisted living facility*.’ The possible answers were: ‘*(a) You don’t think that is an option*. *(b) You talk this through with your partner*. *(c)You ask your mother in-law to consider this*.’ In this case, we also asked participants to assess what they would think other members of their community would do, when faced with the same scenario. We expected those dimensions that are central at the Community level (i.e., Honor, Hierarchy, Loyalty) to be related to a prioritization of group over individual interest. By contrast, we hypothesized that Agency would lead to prioritization of individual over group interests.

We combined the 17 scenarios, after recoding in the ‘group priority’ direction where this was necessary, to one mean variable ‘Scenarios’. We then performed three separate sets of multiple regression analyses, testing four models: the first model (and the first step of all three analyses) specified the cultural identity categories as sole independent variable category. With the second model, we added ComCol (mean of communal levels of Loyalty, Honor and Hierarchy) plus Communal Agency (Com_Ag) in the second step, whereas in the third model PerCol (mean of personal levels of Loyalty, Honor and Hierarchy) and Agency at the personal level (Per_Ag) were added. Finally, in the fourth model we added both ComCol and PerCol, as well as Agency at both levels. The results are shown in Table 8.

**Table 8:** Regression analysis: Behavioral intentions predicted by cultural identity and CCS.

	Model 1		Model 2		Model 3		Model 4	
	Regression Coefficient (Standard Error)	Standardized Coefficient	Regression Coefficient (Standard Error)	Standardized Coefficient	Regression Coefficient (Standard Error)	Standardized Coefficient	Regression Coefficient (Standard Error)	Standardized Coefficient
**Cultural identity**								
Protestant	.097 (.031)	.166**	.055 (.032)	.096	-.050 (.031)	-.085	.040 (.032)	.069
Turkish	.205 (.034)	.321**	.135 (.036)	.211**	.083 (.033)	.128*	.120 (.037)	.184**
Hindustani Sur.	.186 (.031)	.318**	.126 (.033)	.215**	.098 (.031)	.166**	.128 (.034)	.217**
**CCS**								
ComCol			.095 (.020)	.230**			.056 (.030)	.134^†^
Com_Ag			-.038 (.018)	-.100*			-.006 (.020)	-.016
PerCol					.123 (.020)	.275**	.078 (.029)	.178**
Per_Ag					-.062 (.018)	-.153**	-.060 (.021)	-.148**
R^2^ (Δ)	.10**		.16**(.053**)		.19**(.093**^)^		.20**(.094**)	
N	458		433		442		420	

*Note*: Cultural identity represented as three dummy variables with non-religious Dutch as reference group; ComCol (mean of Honor, Loyalty and Hierarchy at Community level), PerCol (mean of Honor, Loyalty and Hierarchy at Personal level), Com_Ag (Agency at Community level) and Per_Ag (Agency at Personal level) centered at their means.; ΔR^2^ is expressed relative to Model 1.

^†^: .1< p < .05

*: p < .05

**: p < .01

What becomes apparent, is that in all cases the addition of CCS(-sub scales) leads to a significant rise in explained variance: both ComCol and PerCol predict behavioral intentions. The addition of CCS does not fully mediate the behavioral intentions for Turks and Hindustani Surinamese, but it does for the protestant Dutch. As expected, values we consider central to the community (Loyalty, Hierarchy, Honor) are related to greater prioritization of group interest, whereas Agency is related to prioritization of individual interest. Results also revealed that behavioral intentions with regard to the scenarios are best predicted by the values at the personal level (PerCol and Per_Ag). This is not wholly unexpected as most scenarios (13 of the 17 scenarios we administered) were concerning individual behavioral intentions. We therefore separately combined and analyzed the individual scenarios (13) and scenarios that concerned the behaviors of other community members (four scenarios, see Table 9). The analyses confirmed that in scenarios in which participants were asked to estimate what their family members would do, values at the communal level were more potent predictors. This is in line with our predictions that people judge the actions of their community members on the basis of what they perceive as the values of that community and not per se on the basis of their personal values.

**Table 9:** Regression analysis: Individual behavioral intentions and those of other community members predicted by cultural identity and CCS.

	Behavioral intentions of other community members		Own behavioral intentions	
	Regression Coefficient (Standard Error)	Standardized Coefficient	Regression Coefficient (Standard Error)	Standardized Coefficient
**Cultural identity**				
Protestant	.009 (.049)	.011	.044 (.037)	.066
Turkish	.257 (.057)	.267**	.096 (.043)	.129*
Hindustani Sur.	.065 (.052)	.075	.154 (.039)	.230**
**CCS**				
ComCol	.111 (.046)	.179*	.042 (.035)	.088
Com_Ag	.051 (.032)	.087	-.019 (.024)	-.043
PerCol	-.004 (.045)	-.007	.097 (.034)	.193**
Per_Ag	-.051 (.033)	-.086	-.064 (.025)	-.139**
R^2^	.13**		.17**	
N	419		420	

*Note*: Cultural identity represented as three dummy variables with non-religious Dutch as reference group; ComCol (mean of Honor, Loyalty and Hierarchy at Community level), PerCol (mean of Honor, Loyalty and Hierarchy at Personal level), Com_Ag (Agency at Community level) and Per_Ag (Agency at Personal level) centered at their means.

*: p < .05

**: p < .01.

In order to further assess differences between groups with respect to loyalty towards extended family, we used a scenario in which the respondent’s grandparents were advised (by a doctor) to admit themselves to residential nursing care. Note that in this case (Sample 1) we only assessed differences between Dutch and Turkish Dutch. Participants were then presented four items regarding their attitudes towards formalized care (which we considered to be the more individualistic approach) versus care by family members (considered to be the collectivist approach). These items were combined in a scale reflecting endorsement of family care (e.g., ‘I think it is a disgrace if elderly people are placed in a nursery home while they still have family that could provide care’; α = .74). Item scores ranged from 1 (totally disagree) to 5 (totally agree). Analysis of variance revealed significant effects such that participants of Turkish (M = 3.07, SD = .76) compared to those of indigenous Dutch descent (M = 2.38, SD = .66) showed higher endorsement of family care, *F* (1, 389) = 91.76, *p* < .001; partial η^2^ = .19. We then determined whether the attitudes ranging from endorsement of formalized care (low score) to endorsement of family care (high score) can be significantly predicted by CCS, using regression analysis. We entered ComCol, PerCol and Agency at both levels as independent variables. ComCol, PerCol, Com_Ag and Per_Ag explained about 27% of the variance (*R*^*2*^ = .27, *F*(4,388) = 16.32, *p* < .001). For a direct comparison, we also examined to what degree INDCOL95 predicted responses on this scenario. The INDCOL95 sub-dimensions (Horizontal Collectivism, Vertical Collectivism, Horizontal Individualism and Vertical Individualism) explained considerably less variance: *R*^*2*^ = .07, *F*(4,385) = 7.31, *p* < .001).

We can conclude that CCS predicts behavioral intentions well. For one, ComCol and PerCol predict behavioral intentions that prioritize group interests, whereas Personal and Communal Agency predict prioritizing personal interests. Moreover, communal level scores better predicted scenarios regarding behavior of community members, whereas personal level scores better predicted behavioral intentions in scenarios that related to individual level behaviors. Taken together these results further underline the importance of measuring values central to communities (and the individual) both at the community and the personal level.

### Does CCS predict actual behavior over time?

In order to consider whether CCS predicts actual behavior over time, we re-sampled a population 1.5 years after they had completed the CCS scale as part of an unrelated study on collective action (in relation to CCS). Since measuring CCS at time 1 (March 2014), there had been national elections in Turkey (June 2015) and we wanted to assess whether CCS (measured 1.5 years beforehand), would affect voting behavior, as a means of affirming predictive validity of CCS. Note that Turkey is a good country to conduct such a study since it contains a mixture of modern and traditional subgroups which are divided not just geographically (e.g., city vs. countryside) but also politically. We predicted that higher scores on Loyalty, Hierarchy and Honor (as expressed in the means ComCol and PerCol) would predict voting for one of the conservative parties. The rationale behind this expectation was that higher scores on ComCol and PerCol would constitute more traditional community values, and since these traditional values were espoused by the conservative AK party of Erdogan, this should result in voting conservatively.

In November 2015, we asked participants if, and for what party, they had voted in the Turkish national elections of June 2015. 111 participants (M_age_ = 39.4, 49 women and 62 men) took part both at time 1 (March 2014; measurement of CCS) and time 2 (November 2015, measurement of voting behavior). Participants were provided with a choice between the five main parties (Ak Parti, CHP, MHP, HDP and SP/BBP, for which 79% of the sample had voted) or for ‘other’ parties, not voting and blank voting (21%). We expected CCS to predict a choice for a Conservative (Ak Parti, MHP and SP/BBP) vs. Progressive party (CHP and HDP). We recoded the voting behavior into a dichotomous variable. Voters who did not vote, or did not vote for a conservative or progressive party were considered ‘missing’ values (n = 23).

We conducted a logistic regression analysis with the (dichotomous) voting behavior as the dependent variable and ComCol, PerCol, Com_Ag and Per_Ag as predictors. A test of the full model against a constant-only model proved statistically significant (χ^2^ = 12,444, *p* = .014, df = 4). The overall explained variance was (Nagelkerke’s) R^2^ = .177, while ComCol proved the only significant predictor (Wald = 4.849, *p* = .028). The odds ratios, Exp(B) = 3.265, indicated that a one point higher score on ComCol raises the chance the respondents vote for a conservative party more than threefold, confirming our predictions.

Importantly, the present study reveals that CCS can predict voting behavior over time–behavior that is quite consequential also at the national level. Moreover, this study once again affirms the ability of CCS to predict within country differences such as a preference for conservative versus liberal parties.

### How does CCS relate to social networks?

Differences in attitudes towards one’s family group are one of the central distinctions between collectivistic and individualistic cultures [34]. Family is considered a more integral part of one’s group and self-concept in collectivistic compared to individualistic cultures. We therefore assessed to what extent CCS can predict attitudes with respect to social networks (Sample 1). We expected that higher CCS scores would be related to feelings of proximity towards family but not friends or colleagues. This is consistent with the group differences we also expected to find: Turkish group would report more proximity towards family, whereas the Dutch were expected to report more proximity with non-relatives (as well).

Participants responded to the question (‘*State for the following people how close they are to you*: *mother; father; brother; sister; grandparents; life partner; best friend; friends; colleagues; boss*’) by moving a slider between 1 (‘Very distant’) and 10 (‘Very close’). The items ‘mother’, ‘father’, ‘brother’, ‘sister’ and ‘grandparents’ were combined into a new measure of perceived Closeness to Kin, while ‘life partner’, ‘best friend’ and ‘friends’ were combined into Closeness to Friends, and ‘colleagues’ and ‘boss’ were combined into Closeness to Work, thus delineating three segments of social network. Participants were asked to fill in an answer even when the question did not fully apply to them. They were asked to imagine what they would think in the given situation.

We found expected group differences such that Closeness to Kin was significantly higher for the Turkish group, while Closeness to Friends was significantly higher for the Dutch group. For Closeness to Work there were no group differences. More importantly, a hierarchical regression analysis shows that CCS significantly predicts Closeness to Kin: *R*^*2*^ = .22, *F*(8, 382) = 13.49, *p* < .001, and the addition of the dummy-coded cultural group to the model does not significantly add to the amount of explained variance. In other words, Community Collectivism explains the bulk of the differences between cultural groups on the topic of closeness to family members. With respect to Closeness to Friends, CCS dimensions predict *R*^*2*^ = .07, *F*(8,382) = 3.70, *p* < .001, while the addition of cultural group slightly adds to the amount of explained variance (Δ*R*^*2*^ = .045, *p* < .05). For Closeness to Work, the proportion of variance explained by CCS is slightly smaller: *R*^*2*^ = .05, *F*(8,382) = 2.71, *p* < .01. Adding cultural group accounts for an additional Δ*R*^*2*^ = .042 (*p* < .05). We conclude that Community Collectivism is a good predictor of closeness to social relations within one’s personal network.

### Value discrepancy: Are there differences between the Community and Personal levels?

There is reason to believe that individuals might report differences between their individually held beliefs and the beliefs and values of others in their culture (cf. [1]). We were interested in exploring these differences: experienced discrepancies between ones personally held beliefs and those perceived within one’s community might induce a ‘value dissociation’ and be related to lower levels of well-being.

We found differences between responses on Community and Personal levels across all cultural groups in samples 1 and 2. Interestingly, although we find an overall C-P discrepancy, there are considerable variations in the magnitude of this discrepancy depending on the cultural group (Table 10). The general difference across all four dimensions, termed Value Discrepancy, is not significant for indigenous groups, whereas they are for the Turkish and Hindustani-Surinamese groups. In the latter two groups, which are traditionally considered to be more interdependent, the communal level of Hierarchy and Honor are perceived as significantly higher than the individual levels. However, there is a different C-P discrepancy for the dimension of agency: in *all* cultural groups individuals consider themselves to be more ‘agentic’ than their community.

**Table 10:** Value discrepancy: CCS differences between Community and Personal levels.

	Communal level minus Personal level				
Cultural Identity	Loyalty	Honor	Hierarchy	Agency	Value Discrepancy
Non-religious	-.11*	-.04	.08	-.31**	-.04
Protestant	-.15**	.07	-.06	-.45**	-.05
Turkish	-.05	.19**	.29**	-.34**	.13*
Hindustani Sur.	-.03	.22**	.45**	-.60**	.21**
Full sample	-.09**	.10**	.17**	-.43**	.05*

*Note*: N = 465

*: p < .05

**: p < .01; Value Discrepancy: Community level score minus Personal level score.

In order to explore whether the discrepancy between personal and community values is experienced as problematic, we considered correlations between Value Discrepancy, life satisfaction [69] and self-esteem [70]. Unexpectedly, there were no significant correlations with life satisfaction. In addition, self-esteem correlations with difference scores tended to be inconsistent and small (even though the large sample size meant that some were statistically significant). In sum, the results show that the participants can and do distinguish between individual and communal values, which implies that intersubjective measures are qualitatively distinct from personal level measures. However, dissociations between the two levels have no straightforward association with life satisfaction and self-esteem.

## General discussion

The main aim of this research was to develop a reliable operationalization of culture that would differentiate between different cultural groups, and predict attitudes and behaviors. As our results reveal, out of a large pool of items we developed a relatively compact scale, the CCS, which meets all these criteria.

We built our scale on the premise that culture is maintained within people’s social networks and the communities that people are part of. These communities, we argued, shape both the types of values people adhere to (i.e., values that are relevant to and maintain communities), as well as determining the level at which culture should be measured (i.e., at the individual and community level). Taking a group dynamic approach, we identified three processes that serve to uphold long-term (group) relationships: the maintenance of group loyalty, of a within-group hierarchy, and of a system of honor by which group members may regulate adherence to certain standards and norms. We also expected individuals to be motivated to maintain a level of personal autonomy vis-à-vis their communities. For this reason, the fourth value assessed was agency. Importantly, we assessed these values at both at the individual and the Community level in four separate samples.

Analysis of scale factor structure confirmed the hypothesized two-level, four-factor structure. Three factors (loyalty, hierarchy and honor) were found to be highly correlated, and could be considered components of a general factor related to group dynamical mechanisms of social regulation—collectivism in other words. The fourth factor, agency (more closely related to individualism), was separate. Each of these factors has good psychometric properties and adequate reliability. This is particularly important because other measures of individualism/collectivism have not always achieved this objective.

The results further showed that CCS could detect sizable differences between cultural groups, both at the Communal and the Personal level. Differentiation between cultural groups was very good in particular for the collectivist dimensions Hierarchy and Honor, as well as for Loyalty. These differences were shown for direct comparisons between indigenous Dutch versus Turkish Dutch (Samples 1 and 2), and between indigenous “secular” Dutch, indigenous strict-protestant Dutch, Dutch of Turkish descent and of Hindustani-Surinamese descent. Notably, the between-group differences tended to be descriptively larger at the Communal level than at the Personal level. There was one exception to this: for orthodox Christians, collective level effects were somewhat smaller, possibly because these communities are more integrated with secular Dutch communities. We conclude that, across the board, Community Collectivism is better at detecting between-group differences than the personal-level measure of collectivism.

Furthermore, the dimensions of Community Collectivism not only differentiate between groups that are generally considered to be very different cultures (e.g., Turkish, Hindustani-Surinamese and native Dutch), but also between other subcultural groups within Dutch society which are more subtly different (strict-protestant indigenous Dutch). This suggests we managed to measure cultural values with a relatively high degree of precision.

By contrast, the agency dimension (which is more individualist in nature) did not differentiate between cultural groups in some instances. This is an intriguing finding: it suggests that one of the reasons why previous work on individualism/collectivism may have struggled to find robust between-culture differences is that it failed to differentiate among psychometrically different dimensions of Ind/Col. It also confirms prior suggestions that individualism and collectivism should not be considered opposite ends of one continuum, but should instead be studied as separate dimensions [44, 34].

Finally, CCS at the communal and personal level predicted behavioral intentions as well as actual behaviors. We tested behavioral intentions and attitudes with a range of scenarios touching on themes such as medical and psychosocial healthcare, family values, and personal freedom, and tested behaviors with regard to voting. Across these themes, CCS boosted the explained variance significantly or proved a potent predictor by itself. More specifically, communal level scores better predicted scenarios regarding behaviors of community members, whereas personal level scores better predicted behavioral intentions for scenarios in which individual behaviors were targeted. At both levels, our scale was a stronger determinant of these intentions than the reduced INDCOL95.

Importantly, CCS also proved a potent predictor of behavior over time: CCS predicted voting behavior 14 months after it was administered. Indeed, a 1 point higher score on ComCol was found to raise the chances of voting for a conservative part by threefold. These findings also speak to CCS’ ability to predict within country differences in actual behavior. We feel this provides strong evidence for the predictive validity of CCS.

In sum, CCS (consisting of two subscales ComCol and Percol) is a valuable and distinctive empirical contribution. The empirical value of CCS becomes apparent in comparison with established measures of individualism/collectivism such as INDCOL. This measure has raised various methodological concerns of reliability, measurement invariance and (content) validity (e.g., [9, 16, 71–73]). Notably, it has been suggested that validity of INDCOL may be improved by “situating” the measure in a specific context or reference group (e.g., [65, 74]). The current findings support this suggestion. Additionally, the content of CCS (i.e., the 4 dimensions) are closely tied to the functions of these values for the community. In this way, CCS extends and refines the concepts of Individualism and Collectivism, as evidenced by the convergent validity with INDCOL95. More precisely, the value domains of loyalty, hierarchy and honor, are mechanisms that regulate the interests of the group, whereas the value domain of agency is aimed at regulating the margins for individuality. These two axes are similar to what Kağıtçıbaşı (e.g., [44]) calls relatedness and autonomy. The combination of content and form of CCS makes that, compared with INDCOL’95, CCS has superior reliability, superior ability to differentiate between cultural groups and superior predictive validity, at the personal level but particularly at the Community level.

### Theoretical implications

The central conclusion of the empirical work is that CCS is a reliable operationalization of community-based cultural differences, as well as an effective predictor of behavioral consequences. The empirical effectiveness of CCS stems from the conceptual analysis that underpins it, we believe. CCS is not just another attempt to operationalize cultural values. CCS operationalizes what we see as the *twin* core ingredients of the cultural process within communities: the values and cultural preferences within individual minds (as operationalized by many others, e.g., [8]) matter as much as the social dynamics between community members within which they develop, are activated and expressed (this paper).

*A distinctive approach to intersubjectivity*. CCS operationalizes this cultural dynamic by tapping into community members’ perceptions of others’ values and practices. In this, our approach builds on the intersubjective approach to culture [1, 75–77] and further corroborates the merits of this approach. Our research does shed some new light on the issue at what level intersubjective norms are best measured. Fischer [17] has shown that the consensus with regard to most intersubjective norms tended to be fairly low—so low in fact that it seemed unjustified to claim that these are shared representation of values. But most work on intersubjective norms has hitherto examined norms that are shared *nationally*. The present work not only shows that the operationalization of intersubjectivity within community settings has merit, we are also able to explain why there is strong *within-nation* difference between cultural groups: We are able to demonstrate large differences between subcultural groups (with a median effect size of *d* = 1.02). CCS was also able to detect differences between ethnic Dutch groups with different community structure (due to religious orientation) and within Turkish society, where there is a long-standing division between traditional and secular lifestyles that can be witnessed in many other Muslim societies. The presence of sizable between-community differences suggests that it may be fruitful to not just focus on the Nation or on large social categories, but rather to operationalize culture at the level at which it is *done*: within smaller, more homogeneous, subcultural communities.

Our focus on the social dynamics within communities also informed what values we expected to be most influential within the community context. Most intersubjective approaches to cultural values have translated the idea of shared or communal meaning into a distinction into different types of reference groups (e.g., the self versus groups people think are central in their culture, [5]). We propose that a consequence of intersubjectivity is that we need to measure those values that are likely to be functional at the level at which they are shared and operate. Thus, cultural values that are central to intragroup processes in close-knit communities (i.e., Loyalty, Hierarchy, Honor), should be recognized to exist at exactly that Community level and should (as our results confirm) predict cultural differences and behavior within those communities. It is for future research to test the corollary of these predictions, that these cultural values are less clearly defined at other levels of abstraction (such as the national level) and that they fail to predict behaviors outside of the community context. Moreover, future work on intersubjectivity may adopt a similar approach to ours by asking what types of values would be most relevant to National groups, social categories or groups at any other level of social abstraction.

*A distinctive approach to values*. CCS assumes that cultural values fulfill a function for the community. In this, our approach builds on Schwartz’ idea that values have functions for individual, biological as well as social reasons (e.g., [3]). But Schwartz approaches this issue from the perspective of the individual: it builds a catalogue of possible values that an individual can hold. Our focus is more situated and focused on the systemic characteristics and dynamics within communities. This leads to marked differences in operationalization: CCS explicitly defines the ‘social other’ as an ingroup member and incorporates only the values and practices directly relevant to the cultural (group) process. Moreover, CCS operationalizes these concepts intersubjectively. CCS is thus different conceptually *and* empirically; there is limited overlap with Schwartz’ SVS.

To illustrate this with some examples from the scales themselves, the SVS asks respondents to rate values (e.g., honouring of parents and elders; showing respect) for their importance “as a guiding principle in my life”. CCS measures values by reference to the community as well as the individual (e.g., item #2 for measuring honor at the community level: ‘In my community, honor is the most important thing for people’). But CCS also measures the practices and beliefs through which these values are maintained (e.g., items 3 and 4 of the same subscale: Our community monitors if people observe the unwritten rules; In my community, members of the family feel responsible for preserving and protecting another family member’s honor). In all, this makes CCS a scale which is more descriptive of how hierarchy, honor, loyalty and agency are *done* within the *community*. By contrast, Schwartz’ approach is most interested in how individuals see *themselves* across a broader circle of values.

*Applications of CCS*. Beyond the ability to distinguish between cultural groups, the present research offers some perspectives on possible applications. One of these was that we had expected and hoped to use CCS as a method for examining value conflict. Several previous studies have found a discrepancy between personal and cultural values (cf. [1]). The current research also finds highly variable differences between communal and personal levels (ranging from large to almost zero), and we expected these discrepancies to be meaningful and consequential; we measured self-esteem and life satisfaction to explore this. But on this point the results were clear, consistent and negative: the magnitude of discrepancy between personal and social values was largely unrelated to measures of life satisfaction and self esteem. Future research might delve deeper into the meaning and consequences of these discrepancies. It could be that they only become problematic in contexts where value discrepancies produce actual relational conflicts, but that integrating the complexities of such discrepancies is a task that humans are normally well equipped for (cf. [78, 79]).

With respect to the implications of CCS for within- and cross-community behavior, there are several practical implications of this research. It is rather obvious that CCS should regulate behavior within communities—future work should certainly focus on the ways in which ComCol and PerCol interact in doing so. But our scenarios were also designed to probe the consequences of community collectivism for cross-community behavior. In particular, we designed scenarios to speak to problems of intercultural adaptation and communication within *healthcare settings*. To give one concrete example: in one of our scenarios, participants were asked to consider whether they would ask their mother in-law, who recently lost her spouse and who lived with the participants’ family from that moment on, to move to a home for the elderly. We found that the behavioral intentions for this scenario were strongly predicted by CCS (both at Community and Personal levels) because this scenario presents a dilemma between the within-community collective demands of certain groups and the incompatible services offered by the Dutch healthcare system. Our assessment of behavioral intentions in this domain confirms that the value that certain cultural minority groups attach to their community and, more specifically, to honor, loyalty and hierarchy, may prevent them from accepting healthcare services. This may help healthcare professionals understand why certain cultural groups consistently underuse certain healthcare services (which in the Netherlands are paid for by *every* taxpayer and thus should be equally accessible to all). More broadly, our scale can inform thinking about systemic interventions, taking into account norms and values of cultural communities as well as the choices of individual clients.

Finally, the behavioral implications of CCS for voting behavior in Turkey point to another implication. The basic values cultivated within communities may be politically consequential at the national level. We confirmed that higher scores on CCS, specifically the domains of ComCol (Loyalty, Hierarchy and Honor) predicted voting for conservative parties. This we explain by the fact that the collectivistic ‘family values’ within communities are propagated, in Turkey, by several conservative parties. But although a priori we would have predicted both components of CCS to be good predictors, empirically ComCol was a stronger predictor. The implication is clear: political parties may profit from knowing the relational dynamics within communities: the values and practices within them might be as relevant (or more) than the individual values of voters. More broadly, this result points to new avenues for cross-cultural research: intra-cultural variability (in the form of between-community differences within the same overarching culture) is predictive of consequential forms of individual behavior directed at the national level.

### Limitations

Although we were able to predict attitudes towards social networks such as family, friends and work as well as behavioral intentions regarding for example formalized healthcare, not all of the scenarios we used to assess differences in cultural behaviors showed significant effects. We think that the effect sizes in tests of predictive validity were substantially reduced by the fact that participants were presented with three choices, of which the middle option, which was generally nuanced and common sense, proved the most appealing for the large majority.

We also note that the studies reported in this paper, although focusing on a wide range of different cultural groups, assess the scale in one language and one overarching cultural setting (the Netherlands). The paper does report one study conducted in Turkey which shows the scale to be useful in that context and language, too. Moreover, although not reported in this paper, the Turkish translation of the scale has a similar factor structure and we are hopeful that its validity can be generalized across other cultural groups and languages in the future.

Relatedly, the measurement invariance of the scale should be tested further. The Community level scale demonstrated scalar invariance, meaning that it is meaningful to compare the means on this scale across subcultural groups. The Personal level scale met all criteria for the metric level of invariance, two of the criteria for scalar invariance (RMSEA and the χ^2^/df-ratio) but not the third criterion (decrease of Comparative Fit Index). Given the fact that CCS consists of both, we think that measurement invariance of CCS was largely confirmed. However, it is possible that measurement invariance can be further improved in future research, possibly by adding or changing items at the Personal level.

## Conclusions

The Community Collectivism Scale provides a means to meaningfully and reliably discriminate between subcultural groups. We provided evidence that the CCS can explain part of the inter-cultural variance in particular behavioral choices, and thus conclude that the CCS allows us to better study inter-cultural variability. These cultural values are of immediate relevance for the regulation of the behavior of individuals in their direct community environment. Importantly, the content of these familial values is also relevant for societal values, and as such relate to how individuals and communities think that society as a whole should function. The present research suggests that CCS will prove to be a fruitful lens through which to understand differences in community values both within and between cultures. But more broadly, we believe that the empirical effectiveness of CCS offers a different perspective on the importance of studying the cultural dynamic within communities: the objective should be to study culture as it manifests itself in individual minds as well in tandem with how culture is *done*. In sum, CCS has much to offer to the fields of cultural and cross-cultural psychology.
